# An Experimental Test of Central Place Foraging Theory in a Cooperatively Breeding Bird

**DOI:** 10.1002/ece3.72697

**Published:** 2025-12-16

**Authors:** Grace Blackburn, Elizabeth M. Wiley, Alex Thompson, Amanda R. Ridley

**Affiliations:** ^1^ Centre of Evolutionary Biology, School of Biological Sciences University of Western Australia Perth Western Australia Australia; ^2^ Coastal Marine Ecosystems Research Centre Central Queensland University Rockhampton Queensland Australia; ^3^ FitzPatrick Institute of African Ornithology University of Cape Town Rondebosch South Africa

**Keywords:** central place foraging, cooperative breeding, parental care, pied babbler, provisioning

## Abstract

Central place foraging, whereby individuals are constrained in their foraging by their need to return to a central place, is common in nesting birds. A key prediction of the central place foraging theory models developed by Orians and Pearson is that as individuals forage further from their central place, optimal load size will increase in order to maintain the same energetic profitability. While this prediction has received widespread support, to our knowledge, it is yet to be tested in a cooperatively breeding species in which multiple adults care for young. In this study, we experimentally tested this central place foraging theory prediction in wild cooperatively breeding Southern Pied Babblers ( *Turdoides bicolor* ) by supplementally feeding individuals with different numbers of mealworms (load sizes of 1, 2 or 3 mealworms), at different distances from their active nests. While babblers were more likely to return to the nest to feed young when given a larger load and when given food closer to their nest, the probability of returning to the nest to feed young at a greater distance did not differ with load size, thus revealing no support for the load size–distance relationship predicted by Orians and Pearson. However, the likelihood of babblers returning to the nest to feed young was affected by the chick:adult ratio, with birds more likely to provision if there were more chicks per adult individual, and was also influenced by the relationship between age and load size, with older birds significantly more likely to feed young when given three mealworms compared to younger birds given the same load size. Our results represent the first experimental test of central place foraging theory in a cooperatively breeding bird species and highlight the importance of considering sources of variation in provisioning behaviour.

## Graphical abstract

This study experimentally investigated central place foraging theory in a cooperatively breeding bird, the Southern Pied Babbler. We found that while birds were more likely to provision when given food closer to the nest and when given a larger load size, the likelihood of babblers provisioning nestlings when given food further from the nest was not affected by load size.

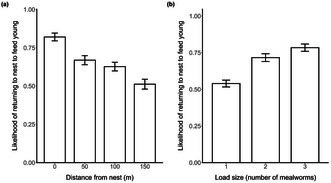

## 1 Introduction

Many species carry food or other resources to a central place rather than consuming it in situ (Woodgate and Chittka 2022). These ‘central place foragers’ include social insects collecting food for their colony (Martin and Vinson 2008; Schmid‐Hempel et al. 1985), birds provisioning nestlings (Carlson and Moreno 1981; Hegner 1982; Kaspari 1991; Martindale 1983), and species that retreat to a burrow or other central place to hide from predators (Bowers and Ellis 1993; Decker et al. 2019). For these animals, foraging time includes not only the time taken to find food but also the time taken to transit both to and from a central place to the foraging patch (Elliott et al. 2009; Orians and Pearson 1979). To maximise foraging efficiency and subsequent fitness, central place foraging theory states that optimal foragers should travel to foraging patches close to their central place via the most direct route (assuming equal foraging patch quality) (Elliott et al. 2009; Orians and Pearson 1979). As the distance between the foraging patch and central place increases, the foraging time will inevitably increase, and therefore the optimal load size (i.e., food brought back to the central place) will also need to increase to make this extended trip energetically profitable (Orians and Pearson 1979). For single prey loaders, this means foragers must locate a larger prey item further from the central place (Hegner 1982; Martindale 1983), while for multiple prey loaders (species that can carry multiple food items at once), this means collecting more food items at more distant patches (Carlson and Moreno 1981; Orians and Pearson 1979).

This prediction of the central place foraging models developed by Orians and Pearson (1979) (that load size should increase with distance from the central place) has been supported by numerous studies on nesting birds (Alonso et al. 1994; Burke and Montevecchi 2009; Carlson and Moreno 1981; Carlson 1983; Elliott et al. 2009; Frey‐Roos et al. 1995; Hegner 1982; Kacelnik 1984; Martindale 1983). For example, white‐fronted bee‐eaters ( *Merops bullockoides* ), grasshopper sparrows (*Ammodramus savanarrum*), and thick‐billed murres ( *Uria lomvia* ) were all found to bring back larger prey items from distances further from the nest compared to closer to the nest (Elliott et al. 2009; Hegner 1982; Kaspari 1991). However, there are also studies in which no relationship between load size and distance from the central place was observed [e.g., female calliope hummingbirds, *Stellula calliope* (Tamm 1989); merlins, *Falco columbarius* (Sodhi 1992)], and recent studies have criticised these central place foraging models as oversimplifications of natural situations (Olsson et al. 2008; Woodgate and Chittka 2022). Furthermore, while some studies report significant interindividual variation in provisioning behaviour (Kaspari 1991; Martindale 1983; Tamm 1989), they generally do not take into account factors that may be behind this variation—such as adult age, brood size, or individual foraging abilities, which may affect foraging and movement decisions and behaviour (Carlson and Moreno 1981; Carlson 1983; Hegner 1982; Martindale 1983; Sodhi 1992; Tamm 1989).

Despite considerable research since the inception of central place foraging theory, no studies to our knowledge have investigated this theory in a cooperatively breeding species in which all group members assist in raising and provisioning young. The foraging and provisioning behaviour of individuals is likely to differ significantly in pair‐living compared to cooperatively breeding species (Ridley and van den Heuvel 2012). This is because, in cooperatively breeding species, there are often multiple adults present to feed young, resulting in less investment in young required per adult (Johnstone 2011; Woxvold et al. 2006). Theoretically, this would mean cooperatively breeding individuals are able to spend more time away from the nest and hence travel further from the central place to find food, which may be beneficial for individuals (particularly those in larger groups) if foraging around the central place leads to prey depletion (Ashmole's halo) (Sorato et al. 2016). Furthermore, foraging and provisioning behaviour may differ between dominant and subordinate (helper) individuals (Ridley and Raihani 2008; Ridley 2007; Russell et al. 2008). Therefore, consideration of whether cooperatively breeding birds follow the predictions of Orians and Pearson's (1979) central place foraging theory is needed.

Southern Pied Babblers (*Turdoides bicolor*, hereafter babblers) are cooperatively breeding passerines endemic to the semi‐arid Kalahari region of southern Africa (Ridley 2016). Babbler groups typically consist of a dominant breeding pair and several nonbreeding individuals (hereafter, subordinates), all of whom assist in reproductive attempts, including brooding and provisioning offspring (Ridley and Raihani 2007). In this species, the amount of time dedicated to the brood differs with dominance status and group size (Bourne et al. 2023; Ridley and Raihani 2008; Ridley 2016; Wiley and Ridley 2016), suggesting individual decisions regarding brood investment are flexible. In this study, we experimentally test a core tenet of central place foraging theory in a cooperatively breeding species by investigating factors affecting individual provisioning decisions when returning to a central place (the nest).

## 2 Methods

### 2.1 Study Site and Species

Data were collected at the Kuruman River Reserve (KRR; 26.92289° S, 21.84048° E) in the Northern Cape, South Africa during the austral summers (September–March) of 2008–2012. The KRR is situated along the Kuruman River in the southern part of the semi‐arid Kalahari region, with vegetation characterized as a xeric savanna (Hunt et al. 2024; Soravia et al. 2023).

Southern pied babblers are medium‐sized (60–90 g) passerines that live year‐round in cooperatively breeding and highly territorial groups comprising a dominant breeding pair and subordinates (Nelson‐Flower et al. 2011). Group size in this study ranged from 2 to 9 adults (i.e., individuals > 1‐year post‐hatching). This species has high reproductive skew, with the dominant pair producing approximately 95% of young (Nelson‐Flower et al. 2011). Dominant individuals can be identified from aggressive displays towards subordinates, affiliative displays and mating between dominant individuals, and overnight incubation by the dominant female (Ridley 2016; Wiley and Ridley 2018). Both dominant and subordinate individuals help to raise young, contributing to incubation, brooding, provisioning, and nest defence (Ridley and Raihani 2007).

The study population has been monitored since 2003 and is habituated to the presence of humans (Ridley 2016), allowing for close observation and experimentation. Individuals within the study population are individually identifiable via unique colour ring combinations. Individuals born into the study population are ringed at 11 days post‐hatching, while adult individuals immigrating into the population are trapped once using a walk‐in trap for ringing. Blood samples are taken during ringing for molecular sexing (as pied babblers are sexually monomorphic) (Ridley 2016). Sex is therefore known for most individuals within the population. Age is also known for individuals born into the population. For adult immigrants, it is assumed to be 1 year old at the time of immigration, or 2 years old for immigrating individuals that breed in the first year in which they immigrate (since dispersal and first breeding are rarely recorded before these ages; Raihani, Nelson‐Flower, Golabek, and Ridley 2010; Wiley and Ridley 2018).

### 2.2 Nest Monitoring and Life History Data

Nest monitoring followed the protocol of Ridley and van den Heuvel (2012). Nests were initially located by observing nest‐building, after which they were checked every 2–3 days to determine the start of incubation, as well as hatch and fledge dates. Nests were considered to have failed when adult babblers no longer visited and attended to the nest. Brood size was determined by the number of gapes reaching out of the nest during feeding events, or where this was not visible, using a mirror mounted on the end of a 3.5 m pole to look inside the nest.

### 2.3 Central Place Foraging Experiment

The central place foraging experiment involved trials whereby individuals were given load sizes of either 1, 2 or 3 mealworms ( *Tenebrio molitor* larvae) at either 0, 50, 100 or 150 m from the central place (active nest). Babblers were rarely seen foraging at distances > 150 m from their active nest (Amanda R. Ridley, pers. obs.); hence, 150 m was chosen as the maximum distance at which to conduct trials. Trials were carried out in the morning (5 a.m. to 11 a.m.) and evening (5 p.m. to 7 p.m.), when pied babblers are most active, and to avoid the hottest part of the day, as high temperatures are known to affect provisioning behaviour in this species (Wiley and Ridley 2016). The load size and distance at which food was given were randomised for each trial. The experimenter waited until babbler individuals were at the randomly selected distance from the nest, and then gave 1, 2 or 3 mealworms to the individual. The behaviour of the bird was then observed, and the outcome of the trial was noted as either ‘fed’ if the bird returned to the nest and provisioned the mealworm/s to the nestlings, or ‘not fed’ if they did not. For each trial, individual and group identity were noted, as well as the sex and dominance status of focal individuals, adult group size, chick age (number of days since hatch date), and brood size. We also noted whether groups had dependent young from an older brood from the same breeding season that they were still provisioning, as this may have affected their provisioning decisions towards nestlings.

### 2.4 Foraging Focals

During weekly visits to babbler groups, 20‐min behavioural observations (focals) were conducted to determine individual foraging effort and efficiency. This involved noting all behaviours of the focal bird (including foraging, as well as the number of prey caught and size of each prey item, categorised following Raihani and Ridley 2007). Foraging effort (i.e., proportion of observation time spent foraging) and foraging efficiency (i.e., biomass of food collected per minute foraging, in grams per minute) were then calculated from focals. Foraging efficiency was only calculated for focals in which birds spent ≥ 5 min foraging, to ensure robust measures of foraging efficiency.

### 2.5 Statistical Analysis

Analyses were conducted in R version 4.4.2 (RStudio Team 2022). To determine the factors affecting the probability of babblers returning to the nest to feed young, Generalised Linear Mixed Models (GLMMs) were fitted using the glmmTMB package (Brooks et al. 2017) with a binomial distribution and a logit link function. All models included trial outcome (‘fed’, ‘not fed’) as the dependent variable, and bird and group identity as random terms.

The initial analysis was conducted on 1018 trials on 116 birds from 17 groups. Explanatory variables included in this analysis were load size (a categorical variable of 1, 2 or 3 mealworms), distance to the nest (a categorical variable of 0, 50, 100 or 150 m), adult group size (number of individuals > 1 year old within the focal group), chick age, brood size, the presence of dependent young from an older brood (‘older brood present’; 0 = groups did not have dependent young from an older brood from the same season; 1 = groups did have dependent young from an older brood from the same season), focal bird sex, focal bird age, focal bird dominance status, and the interaction between load size and distance to the nest (to test the predictions of central place foraging theory). We additionally included an explanatory variable of chick:adult ratio, representing the ratio of chicks to adult provisioners in the group, to account for potential differences in the average ‘expected’ provisioning effort of each adult. A second analysis was conducted on a subset of 830 trials (on 89 birds from 17 groups) for which we had foraging effort and foraging efficiency metrics for focal birds. Foraging effort and foraging efficiency for each trial were calculated by averaging the foraging effort and efficiency of every focal in which that individual foraged for over 5 min conducted within 2 months of the trial. Explanatory variables in this analysis were the same as in the initial analysis, with the addition of foraging effort and foraging efficiency. All continuous predictors were scaled for all analyses (centred on the mean and divided by one standard deviation, see Schielzeth 2010). Explanatory variables were checked for correlation with one another prior to inclusion in models, with a variance inflation factor (VIF) or generalised variance inflation factor (GVIF) > 2 for pairs of predictors being considered a high level of correlation (Fox and Monette 1992). No explanatory terms were correlated with each other (all VIF and GVIF < 2).

Sensitivity power analysis (Greenland et al. 2016; Cohen 1988) was performed with the *pwr.f2.test* function in the *pwr* package (Champely 2020) to identify the minimum determinable effects of main effects and two‐way interactions. We assumed a fourfold increase in required sample size to adequately detect two‐way interactions in mixed‐effects models (Leon and Heo 2009). In line with Cohen (1988), we assumed that *f* values of ~0.02, ~0.15, and ~0.35 represented the ability to detect small, medium, and large effect sizes respectively. Results from sensitivity power analysis are presented in the Tables S2 and S6 for the full (*N* = 1018 trials) and subset (*N* = 830 trials) analyses, respectively.

To determine which predictors best explained variation in data patterns, we conducted model selection using Akaike information criterion values (AIC). AIC values for each model were recorded and compared with a null model containing only the intercept and random terms. The model with the lowest AIC value was considered the most parsimonious model, and all models within 2AIC of this top model were included in the top model set. Terms were only considered significant if their 95% confidence intervals did not intersect zero (Grueber et al. 2011; Symonds and Moussalli 2011). If the 95% confidence interval of a term when tested alone intersected zero, that predictor was excluded from additive models (but could still be included in interactions). In cases where two models had similar AIC values, the model containing the fewest terms contributing to the AIC value was considered the most parsimonious (Harrison et al. 2018).

Posthoc comparisons using the *emmeans* function within the emmeans package (Lenth 2019) were used to obtain contrasts between different levels of categorical predictors. For models with significant interactions between continuous and categorical explanatory factors, the *emtrends* function within the *emmeans* package was used to obtain contrasts between different levels of the interaction. *p*‐values adjusted for multiple comparisons via the Tukey method were also calculated using the *emmeans* and *emtrends* functions.

## 3 Results

**FIGURE 1:**
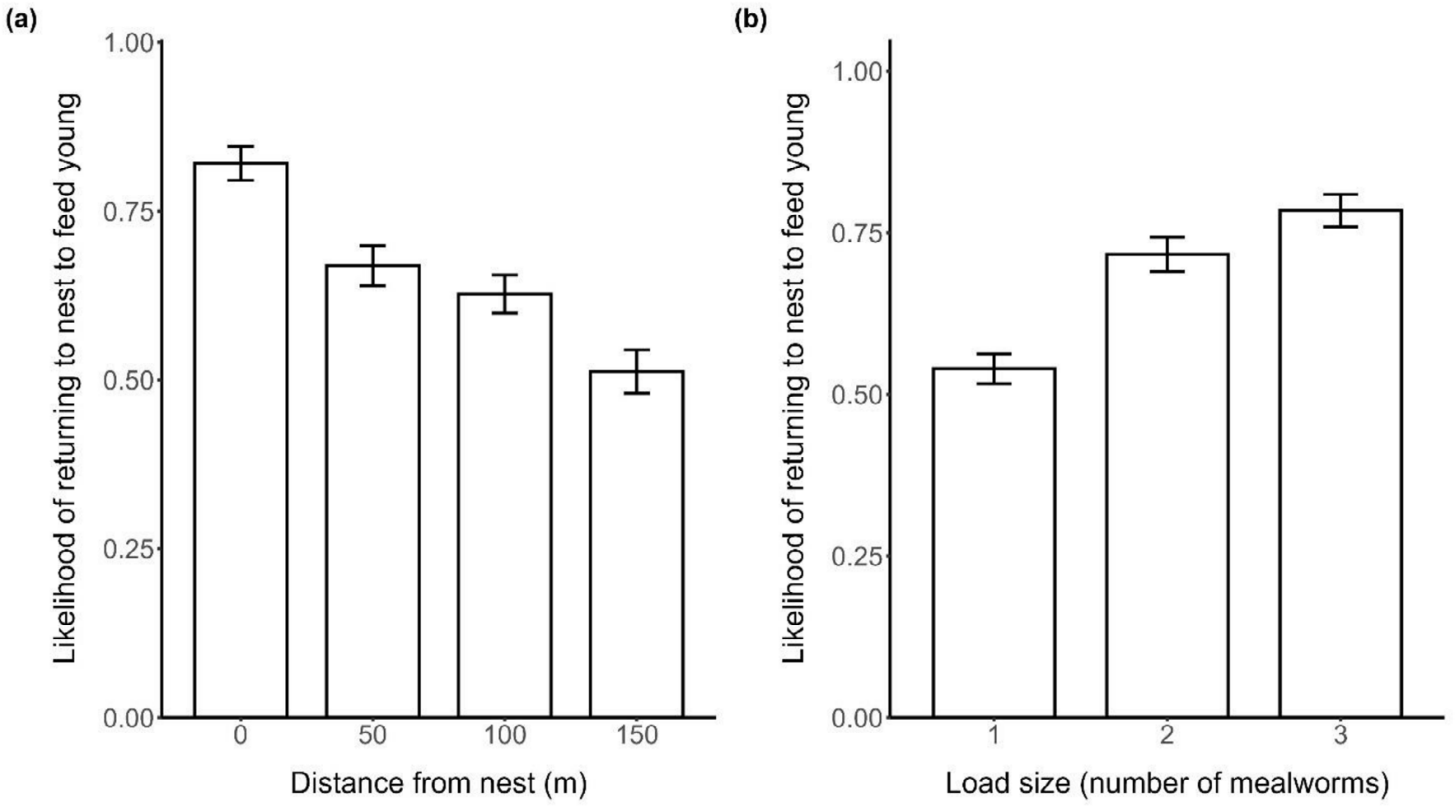
Probability of individuals returning to the nest to feed chicks in relation to (a) distance from the nest, and (b) load size. Error bars represent SEs. *N* = 1018 trials (116 birds from 17 groups).

**FIGURE 2:**
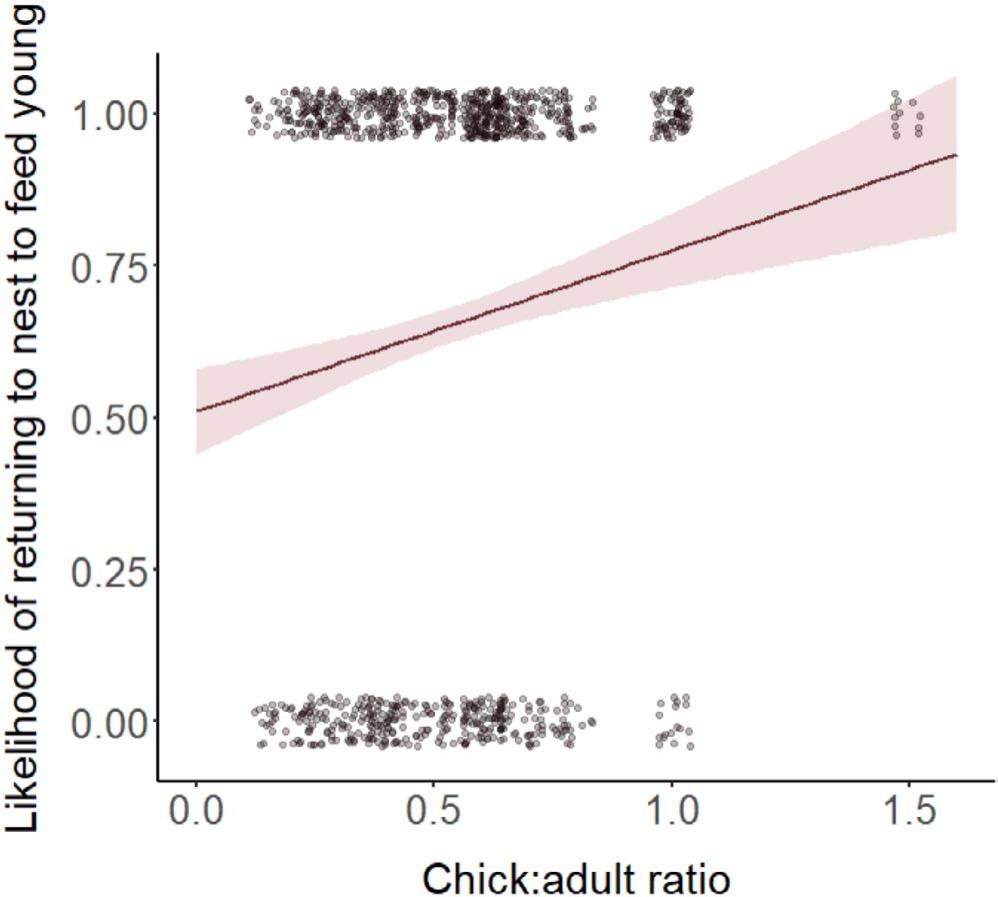
Probability of individuals returning to the nest to feed young in relation to chick:adult ratio. Error bars represent SEs. Points jittered to minimise overlap. *N* = 1018 trials (116 birds from 17 groups).

**FIGURE 3:**
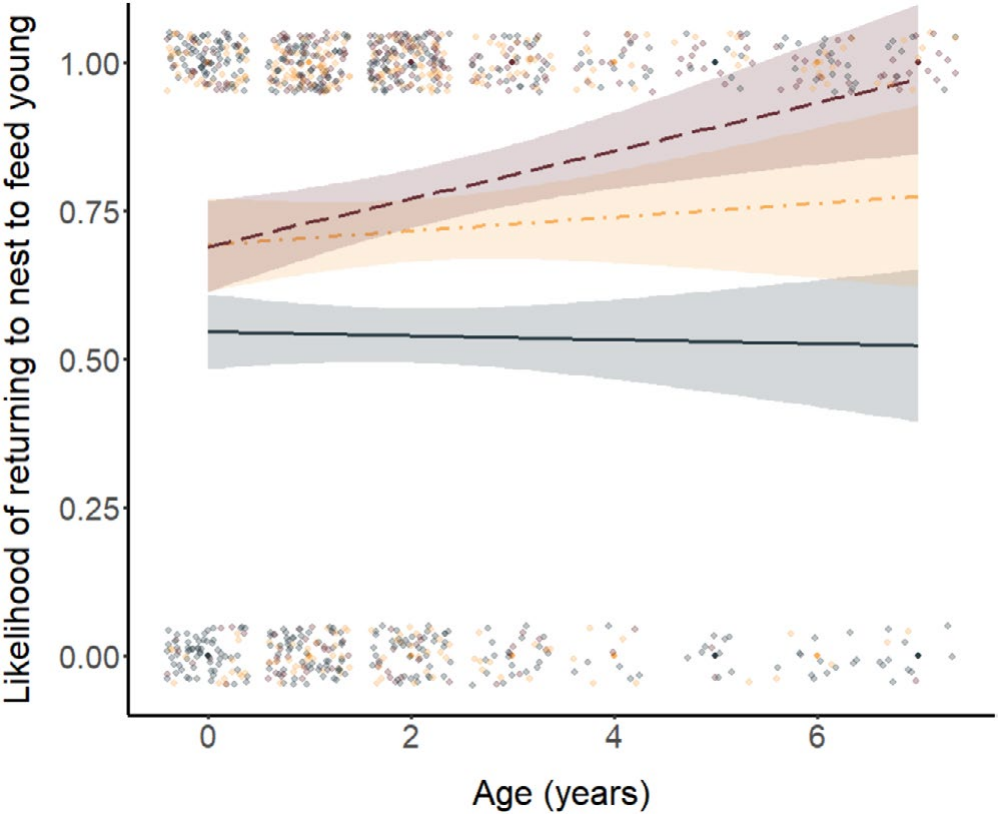
Probability of individuals returning to the nest to feed chicks in relation to the focal birds age and number of mealworms given (grey points and solid line = 1 mealworm; orange points and dot‐dash line = 2 mealworms; maroon points and dashed line = 3 mealworms). Points jittered to minimise overlap. *N* = 1018 trials (116 birds from 17 groups).

Model selection revealed an effect of distance, load, chick:adult ratio, and age on the likelihood of individuals returning to the nest to feed young (Table 1). Birds were significantly more likely to return to the nest to feed chicks if they were closer to the nest compared to if they were further away (Table 1; Figure 1a). The probability of birds returning to the nest to feed chicks dropped from 82.1% at 0 m from the nest to 51.3% at 150 m from the nest. Birds were also more likely to return to the nest to feed chicks if they were carrying a larger load (Table 1; Figure 1b). The likelihood of birds returning to the nest to feed chicks when given 1 mealworm was 54.0% compared to 78.4% when given three mealworms. In addition, individuals were more likely to return to the nest to feed young if there was a higher ratio of chicks to adult provisioners in their group (Table 1; Figure 2). Finally, the interaction between age and load size was also significant, with older individuals significantly more likely than younger individuals to return to the nest to feed chicks when given a large load size (three mealworms, Tables 1 and 2; Figure 3). Post hoc pairwise comparisons of the likelihood of returning to the nest to feed from different distances, and with different load sizes, are provided in Tables S3 and S4.

**TABLE 1:** Top model set of candidate terms affecting the probability of individuals returning to the nest to feed chicks.

Top models	AIC	ΔAIC
Age × load + distance + chick:adult ratio	1150.08	0.00
*Null model*	1286.12	136.04

Parameter	Estimate	SE	CI
Age	−0.01	0.12	−0.25, 0.24
Load			
1 Mealworm	—	—	—
2 Mealworms	1.03	0.19	0.66, 1.41
3 Mealworms	1.39	0.21	0.98, 1.81
Distance (m)			
0	—	—	—
50	−0.98	0.24	−1.46, −0.50
100	−1.21	0.24	−1.68, −0.75
150	−1.97	0.25	−2.46, −1.47
Chick:adult ratio	0.45	0.11	0.24, 0.66
Age × load			
Age × 1 mealworm	—	—	—
Age × 2 mealworms	0.17	0.19	−0.20, 0.55
Age × 3 mealworms	0.70	0.24	0.23, 1.16

*Note:* All models include bird and group ID as random terms. The top model set includes all models within two AIC of the top model and the null model (intercept‐only model) for comparison. Coefficient estimates ± SE and 95% confidence intervals (CI) are given below the top model set. *N* = 1018 trials (116 birds from 17 groups). For a list of all models tested, see Table S1.

**TABLE 2:** Post hoc analyses of the probability of individuals returning to the nest to feed chicks on a subset of trials for which brood size, adult age, sex, and dominance status were known.

Contrast	Estimate	SE	*z*‐ratio	*p*
Age × 1 mealworm	−0.01	0.12	−0.02	0.988
Age × 2 mealworms	0.17	0.17	0.99	0.319
Age × 3 mealworms	0.70	0.22	3.15	0.002

*Note:* Estimates represent the slopes of levels of the interaction between age and load. *N* = 1018 trials (116 birds from 17 groups).

The interaction between load size and distance did not significantly affect the likelihood of individuals returning to the nest to feed chicks (Table S1). Statistical power analyses confirmed sufficient sample size to detect even small effect sizes in two‐way interactions (Cohen's *f* ^2^ = 0.05; Table S2), suggesting that even a small effect size, if present, would have been detected in our analysis. Furthermore, analysis of a subset of trials for which individual foraging effort and efficiency were known revealed no effect of these factors on the likelihood of returning to the nest to feed (Table S5).

## 4 Discussion

The likelihood of babblers returning to the nest to feed young was significantly affected by distance, load size, and chick:adult ratio. Additionally, the effect of load size on the likelihood of provisioning young differed with age, with older individuals more likely to return to the nest compared to younger individuals when given a load size of three mealworms. The interaction between load size and distance was not significant, revealing that the amount of food brought back to the nest did not differ with distance from the nest. We therefore did not find evidence to support Orians and Pearson (1979) central place foraging model for multi‐prey loaders.

### 4.1 Central Place Foraging Predictions

A decreased likelihood of individuals returning to the nest to feed young as food is given further away is likely a means of individuals reducing the energetic costs associated with provisioning (Canestrari et al. 2007; Drent and Daan 1980). Central place foraging theory, and optimal foraging theory more generally, is based on the assumption that individuals should aim to maximise energy gain while minimising time spent foraging (Orians and Pearson 1979; Woodgate and Chittka 2022). It is therefore unsurprising that babblers would be more likely to provision when given food closer to the nest compared to further away, as the relative distance, travel time, and energetic costs of provisioning are lower. Babblers closer to the nest may also be better able to hear the begging calls of chicks [as they are still within the active acoustic space of the begging signals (Brenowitz 1982; Lohr et al. 2003)] and so may be more likely to respond to these signals by provisioning (Mondloch 1995). As birds get further from the nest, the energetic expense associated with flying to the nest increases, while the likelihood of hearing nestling begging calls decreases, which may explain why individuals are less likely to provision at greater distances.

The load size effect identified in this study may similarly be a way of minimising energetic expenditure when travelling to the nest. By increasing provisioning when given multiple mealworms and reducing provisioning when given only one, babblers may be able to minimise the total rate of nest visitation. This could yield two main benefits. Firstly, fewer total visits to the nest means a reduced energetic cost of provisioning (Canestrari et al. 2007; Drent and Daan 1980). Secondly, a reduced nest visitation rate may reduce the likelihood of a predator detecting the nest. The likelihood of a predator detecting a nest will increase with nest visitation rate (Martin et al. 2000; Muchai and du Plessis 2005); therefore, reducing total visitation by provisioning only larger loads may reduce nest predation (Eggers et al. 2008; Strickland and Waite 2001) and subsequently increase brood survival (Raihani, Nelson‐Flower, Moyes, et al. 2010). Furthermore, by self‐feeding when given a smaller load, individuals are able to increase their own energetic intake and hence partially make up for the energetic costs associated with provisioning efforts (Ydenberg 1994).

We did not find a significant interaction between load size and distance in our study; hence, our findings do not support Orians and Pearson's (1979) prediction that central place foragers should increase their load size when travelling to foraging patches further from the central place. There could be several reasons to explain this finding. Central place foraging models have been criticised as oversimplifications of natural situations that ignore environmental stochasticity and individual variation in the ability to optimise foraging (Woodgate and Chittka 2022). In reality, provisioning decisions can be affected by numerous factors (only some of which we were able to control for in this study), including temperature (Wiley and Ridley 2016), rainfall (Trapote et al. 2023), sex (Martindale 1983; Quillfeldt et al. 2004), age (Cauchard et al. 2021), experience (Daunt et al. 2007), predator presence (Mutzel et al. 2013), and the behaviour of other provisioners (Wright and Dingemanse 1999). Furthermore, there is increasing evidence that the provisioning decisions of individuals in cooperatively breeding species are influenced by group members (Canestrari et al. 2007; Halliwell et al. 2022; Koenig and Walters 2016; Raihani, Nelson‐Flower, Moyes, et al. 2010; Trapote et al. 2024; van Boheemen et al. 2019; Wright and Dingemanse 1999). Therefore, central place foraging predictions may not necessarily hold for cooperatively breeding individuals in natural systems, where provisioning decisions are influenced by numerous individual, social, and environmental factors. It is important to acknowledge the possibility that an interaction between load size and distance in a cooperatively breeding system in which many adults provision young may be small relative to what has been found in breeding systems in which only one or a pair of individuals provision young. However, our analysis was conducted on a large sample size with adequate statistical power to detect even small effects (Cohen's *f* ^2^ = 0.05); hence, we can be confident that such effects would have been detected if present. A similar species‐specific explanation for the lack of an interaction effect between load size and distance could be that the range of distances travelled by babblers to and from foraging patches (and hence travel costs associated with foraging) is relatively small when compared to studies that have found support for Orians and Pearson's (1979) central place foraging prediction (Alonso et al. 1994; Burke and Montevecchi 2009; Elliott et al. 2009; Frey‐Roos et al. 1995; Hegner 1982). For those species that travel larger distances to foraging patches, and therefore experience significantly higher energetic costs, it may be more important for individuals to maximise their load size, particularly at distances far from the nest (as predicted by Orians and Pearson 1979).

It is also important to note that the behaviour of babblers during our experimental study may not reflect natural provisioning behaviours. When individuals forage for prey to provision, they must make decisions about how much time to spend in each foraging patch (Carlson and Moreno 1981), when it is more profitable to move to a new foraging patch (Olsson et al. 2008), and the type of prey to bring back (Martindale 1983; Nilsson et al. 2020). In our experiment, babblers were given a pre‐determined amount of a single prey type at a set distance, and hence did not have to make as many decisions as they may be required to under natural scenarios.

### 4.2 Other Factors Affecting Provisioning Behaviour

Babblers were more likely to return to the nest and provision young as the ratio of chicks in the brood to adults in the group increased. For cooperatively breeding species, in which both brood size and the number of helpers can vary, and hence can result in significantly different foraging activity for helpers in different groups (e.g., the nutritional demands of dependent young per helper is higher in a group with a high chick:adult ratio compared to one with a low chick:adult ratio), it is necessary to consider the impact of these measures together on provisioning behaviours. Nevertheless, this aligns with findings across bird species exhibiting biparental care, which have found increased parental care (including nest visitation rate, provisioning rate, and prey size provisioned) in larger broods (Bowers et al. 2014; Cauchard et al. 2021; Sanz 1999; Wright et al. 1998), and is likely reflective of the increased energetic requirements of more nestlings. Interestingly, we also found that the provisioning behaviour of babblers given three mealworms differed with adult age, with older individuals significantly more likely to provision three mealworms than younger individuals. It is important to note that while older individuals are more likely to achieve dominance within a group compared to younger individuals [dominance acquisition on average occurs at 882 days post‐hatching in females and 1085 days post‐hatching in males (Wiley and Ridley 2018)], age and dominance status in this study were not correlated (VIF < 2), and hence we can be confident that this effect was due to age and not dominance status. Several studies across avian taxa have identified increased reproductive success in older individuals (Blas et al. 2009; Daunt et al. 2007; Janiszewski et al. 2017; Włodarczyk and Minias 2020). One of the hypotheses conceived to explain this is that these older individuals are more efficient foragers, and subsequently better provisioners (Daunt et al. 2007; Desrochers 1992). In the present study, we found no relationship between our foraging metrics (foraging effort and foraging efficiency) and provisioning behaviour, suggesting that some other aspect of age accounts for this difference in provisioning behaviour. Older birds may be more experienced in offspring care, having been involved in previous reproductive attempts, and thus may have learnt how to maximise their provisioning efficiency. An alternative explanation for this finding is that younger birds may be prioritising their own body condition over that of the brood, in order to increase their own survival and future reproductive success (Canestrari et al. 2007; Curio et al. 1978).

We found no effect of adult group size on the likelihood of babblers returning to the nest to feed young. The lack of a group size effect identified here supports previous work on this species that found adult provisioning rates did not decrease with increasing group size (Wiley and Ridley 2016). However, our finding that the likelihood of babblers returning to the nest to feed young was affected by the chick:adult ratio suggests that, if the brood size were held constant, the likelihood of an individual provisioning would decrease as adult group size increases. This therefore highlights the importance of considering how brood size and adult group size (or number of helpers) may interact to affect provisioning decisions as well as the benefits of group‐living in cooperatively breeding species.

## 5 Conclusion

Our experimental study revealed that many factors contribute to the provisioning decisions of pied babblers. Load size, distance, chick:adult ratio, and age all affected the likelihood of babblers provisioning young when given mealworms. We did not find evidence to support the prediction that birds should increase their optimal load size with distance from the central place (Orians and Pearson 1979), in line with several other studies that have not found evidence to support this prediction of central place foraging theory (Tamm 1989; Sodhi 1992; Zurbuchen et al. 2010). Our study adds to the body of literature on central place foragers and highlights the importance of considering factors that may affect the provisioning decisions of birds.

## Author Contributions

**Grace Blackburn:** data curation (equal), formal analysis (lead), writing – original draft (lead), writing – review and editing (equal). **Elizabeth M. Wiley:** data curation (equal), investigation (equal), writing – review and editing (equal). **Alex Thompson:** data curation (equal), investigation (equal), writing – review and editing (equal). **Amanda R. Ridley:** conceptualization (lead), data curation (equal), formal analysis (supporting), investigation (equal), methodology (equal), project administration (equal), writing – original draft (supporting), writing – review and editing (equal).

## Ethics Statement

Ethical approval was provided by the University of Cape Town Animal Ethics Committee under ethics number R2012/2006/V15/AR.

## Conflicts of Interest

The authors declare no conflicts of interest.

## Supporting information


**Tables S1–S6:** ece372697‐sup‐0001‐TableS1‐S6.docx.

## Data Availability

All data are available from Figshare online data repository at https://figshare.com/s/7efe09183ef880b55b2e.
