# Supplementary material for: An Experimental Test of Central Place Foraging Theory in a Cooperatively Breeding Bird
Source: Ecol Evol. 2025 Dec 16;15(12):e72697. doi: 10.1002/ece3.72697 (PMC12706523; doi:10.1002/ece3.72697)
Supplement: Supplementary file 1 — Tables S1–S6: ece372697‐sup‐0001‐TableS1‐S6.docx. [file ECE3-15-e72697-s001.docx]

**An experimental test of central place foraging theory in a cooperatively breeding bird**

**Supplementary material**

**Full dataset analysis. N = 1018 trials of 116 birds from 17 groups.**

**Table S1.** Top model set for factors affecting the probability of individuals returning to the nest to feed chicks. Top models are bolded. *N* = 1018 trials (116 birds from 17 groups). Bird and group ID are included as random terms in all models.

| **Predictor** | **AIC** | **ΔAIC** |
| --- | --- | --- |
| **Age * load + distance + chick:adult ratio** | **1150.08** | 0.00 |
| Distance + load + chick:adult ratio | 1156.64 | 6.56 |
| Distance + load | 1174.11 | 24.03 |
| Distance * load | 1181.62 | 31.54 |
| Chick:adult ratio* load | 1217.47 | 67.39 |
| Chick:adult ratio * distance | 1220.11 | 70.03 |
| Sex * load | 1226.52 | 76.44 |
| Age * load | 1227.52 | 77.44 |
| Brood size * load | 1228.50 | 78.42 |
| Brood size * distance | 1230.89 | 80.81 |
| Group size * load | 1231.65 | 81.57 |
| Load | 1232.23 | 82.15 |
| Chick age * load | 1233.70 | 83.62 |
| Dominance status * load | 1233.82 | 83.74 |
| Older brood present* load | 1235.97 | 85.89 |
| Chick age * distance | 1238.22 | 88.14 |
| Age * distance | 1240.34 | 90.26 |
| Group size* distance | 1240.48 | 90.40 |
| Distance | 1240.49 | 90.41 |
| Dominance status * distance | 1243.20 | 93.12 |
| Older brood present* distance | 1244.87 | 94.79 |
| Sex * distance | 1246.09 | 96.01 |
| Chick:adult ratio | 1265.43 | 115.35 |
| Brood size | 1277.79 | 127.71 |
| Dominance status * group size | 1280.13 | 130.05 |
| Group size | 1282.47 | 132.39 |
| Age | 1283.06 | 132.98 |
| Dominance status | 1284.67 | 134.59 |
| Chick age | 1285.50 | 135.42 |
| Older brood present | 1286.10 | 136.02 |
| *Null* | 1286.12 | 136.04 |
| Sex | 1287.68 | 137.60 |

**Table S2.** Power analyses for the main effects and two-way interactions in models. Numerator degrees of freedom are indicated by *u*; denominator degrees of freedom are given by *v*; α indicates the significance level; power indicates the probability of finding a true effect; and Cohens *f^2^* gives the measure of the determinable effect size.

|  | ***u*** | ***v*** | ***α*** | ***power*** | ***f^2^*** |
| --- | --- | --- | --- | --- | --- |
| Main effect | 4 | 1013 | 0.05 | 0.8 | 0.01 |
| Interaction | 5 | 253 | 0.05 | 0.8 | 0.05 |

**Table S3.** Post hoc analyses of the likelihood of individuals returning to the nest to feed from different distances. *N* = 1018 trials (116 birds from 17 groups).

| **Contrast** | **Estimate** | **SE** | ***Z* ratio** | ***P*** |
| --- | --- | --- | --- | --- |
| 0m - 50m | 0.98 | 0.24 | 3.99 | < 0.001 |
| 0m - 100m | 1.21 | 0.24 | 5.12 | < 0.001 |
| 0m - 150m | 1.97 | 0.25 | 7.78 | < 0.001 |
| 50m - 100m | 0.24 | 0.21 | 1.15 | 0.658 |
| 50m - 150m | 0.99 | 0.22 | 4.57 | < 0.001 |
| 100m - 150m | 0.75 | 0.21 | 2.62 | 0.002 |

**Table S4.** Post hoc analyses of the likelihood of individuals returning to the nest with different load sizes. *N* = 1018 trials (116 birds from 17 groups).

| **Contrast** | **Estimate** | **SE** | ***Z* ratio** | ***P*** |
| --- | --- | --- | --- | --- |
| 1 mealworm - 2 mealworms | -1.39 | 0.44 | -3.17 | 0.004 |
| 1 mealworm - 3 mealworms | -2.82 | 0.55 | -5.13 | < 0.001 |
| 2 mealworms - 3 mealworms | -1.43 | 0.61 | -2.35 | 0.049 |

**Subset analysis on trials for which foraging effort and efficiency are known. N = 830 trials of 89 birds from 17 groups.**

**Table S5.** Top model set for factors affecting the probability of individuals returning to the nest to feed chicks. Top models are bolded. *N* = 830 trials (89 birds from 17 groups). Bird and group ID are included as random terms in all models.

| **Predictor** | **AIC** | **ΔAIC** |
| --- | --- | --- |
| **Age*load + distance + chick:adult ratio** | **901.68** | **0.00** |
| Distance + load + chick:adult ratio | 907.92 | 6.24 |
| Distance + load | 929.49 | 27.81 |
| Distance * load | 937.24 | 35.56 |
| Age * load | 981.53 | 79.85 |
| Load | 986.31 | 84.63 |
| Dominance status * load | 986.35 | 84.67 |
| Foraging effort* load | 990.77 | 89.09 |
| Foraging efficiency * load | 991.20 | 89.52 |
| Distance | 991.90 | 90.22 |
| Foraging efficiency * Distance | 996.38 | 94.70 |
| Foraging effort * Distance | 997.36 | 95.68 |
| Chick:adult ratio | 1014.33 | 112.65 |
| Brood size | 1026.46 | 124.78 |
| Group size | 1036.20 | 134.52 |
| Dominance status | 1036.85 | 135.17 |
| Age | 1037.29 | 135.61 |
| *Null* | 1038.03 | 136.35 |
| Older brood present | 1038.46 | 136.78 |
| Chick age | 1038.89 | 137.21 |
| Foraging efficiency | 1039.07 | 137.39 |
| Foraging effort | 1039.65 | 137.97 |
| Sex | 1039.91 | 138.23 |

**Table S6.** Power analyses for the main effects and two-way interactions in models. Numerator degrees of freedom are indicated by *u*; denominator degrees of freedom are given by *v*; α indicates the significance level; power indicates the probability of finding a true effect; and Cohens *f^2^* gives the measure of the determinable effect size.

|  | ***u*** | ***v*** | ***α*** | ***power*** | ***f^2^*** |
| --- | --- | --- | --- | --- | --- |
| Main effect | 4 | 826 | 0.05 | 0.8 | 0.01 |
| Interaction | 5 | 206 | 0.05 | 0.8 | 0.06 |
